# Deciphering microRNA targets in pancreatic cancer using miRComb R package

**DOI:** 10.18632/oncotarget.24034

**Published:** 2018-01-08

**Authors:** Maria Vila-Casadesús, Elena Vila-Navarro, Giulia Raimondi, Cristina Fillat, Antoni Castells, Juan José Lozano, Meritxell Gironella

**Affiliations:** ^1^ Gastrointestinal & Pancreatic Oncology Group, Centro de Investigación Biomédica en Red de Enfermedades Hepáticas y Digestivas (CIBEREHD), Hospital Clínic of Barcelona, Institut d'Investigacions Biomèdiques August Pi i Sunyer (IDIBAPS), Barcelona, Catalonia, Spain; ^2^ Bioinformatics Platform, CIBEREHD, Barcelona, Catalonia, Spain; ^3^ Gene Therapy and Cancer, Institut d'Investigacions Biomèdiques August Pi i Sunyer (IDIBAPS), Centro de Investigación Biomédica en Red de Enfermedades Raras (CIBERER), Universitat de Barcelona, Barcelona, Catalonia, Spain

**Keywords:** microRNA, pancreatic cancer, target prediction, gene expression, CRISPR-Cas9

## Abstract

MiRNAs are small non-coding RNAs that post-transcriptionally regulate gene expression. They play important roles in cancer but little is known about the specific functions that each miRNA exerts in each type of cancer. More knowledge about their specific targets is needed to better understand the complexity of molecular networks taking part in cancer. In this study we report the miRNA-mRNA interactome occurring in pancreatic cancer by using a bioinformatic approach called miRComb, which combines tissue expression data with miRNA-target prediction databases (TargetScan, miRSVR and miRDB). MiRNome and transcriptome of 12 human pancreatic tissues (9 pancreatic ductal adenocarcinomas and 3 controls) were analyzed by next-generation sequencing and microarray, respectively. Analysis confirmed differential expression of both miRNAs and mRNAs in cancerous tissue versus control, and unveiled 17401 relevant miRNA-mRNA interactions likely to occur in pancreatic cancer. They were sorted according to the degree of negative correlation between miRNA and mRNA expression. Results highlighted the importance of miR-148a and miR-21 interactions among others. Two components of the Notch signaling pathway, ADAM17 and EP300, were confirmed as miR-148a targets in MiaPaca-2 pancreatic cancer cells overexpressing miR-148a. Moreover, a CRISPR-Cas9 cellular model was generated to knock-out the expression of miR-21 in PANC-1 cells. As expected, the expression of two miRComb miR-21 predicted targets, PDCD4 and BTG2, was significantly upregulated in these cells in comparison to control PANC-1.

## INTRODUCTION

Pancreatic ductal adenocarcinoma (PDAC) is the fourth leading cause of cancer death in occidental countries and has the worst prognosis of all major malignancies with just a 6% five-year survival rate [1]. By the time of diagnosis, most patients present with locally advanced or metastatic disease that precludes curative resection and have a mean survival of less than 1 year [2, 3]. This fatal scenario is due, in part, to the high aggressiveness of the tumour and the lack of effective treatments. In order to overcome this dire problem, new and more efficient therapeutic targets are urgently needed. To achieve this goal is highly necessary to increase the knowledge about the molecular mechanisms involved in pancreatic cancer and to elucidate intracellular network connections which play indispensable roles for cancer progression.

MicroRNAs (miRNAs) are small endogenous non-coding RNAs of 18-25 nucleotides that negatively regulate gene expression at the posttranscriptional level by either repressing mRNA translation or targeting mRNAs for degradation. One miRNA can modulate up to hundreds of genes and one gene may be regulated by more than one miRNA [4]. MiRNAs are estimated to modulate the translation of more than 60% of protein-coding genes and are involved in regulating a wide range of biological processes such as cellular proliferation, differentiation, apoptosis and development [5, 6]. Their dysregulation plays an essential role in the development and progression of cancer and they can act as tumour suppressors or oncogenes depending on the target that they are regulating in a specific situation [7].

Aberrant expression of miRNAs has been widely reported in human cancers including PDAC [8, 9]. However, the functional meaning of each deregulated miRNA in the context of PDAC is still largely unknown. In order to help with the functional understanding of aberrant miRNomes, we developed an R package called miRComb [10]. This software is able to combine miRNA and mRNA expression data with hybridization information, in order to find potential miRNA-mRNA targets that are likely to occur in a specific context. In this study, we applied miRComb to combine miRNome and transcriptome expression data from human pancreatic cancer tumor specimens, in order to uncover the miRNA-mRNA interactome that is taking place in pancreatic tumorigenesis. The results obtained here will serve to better understand pancreatic tumorigenesis and will help to highlight those miRNA-mRNA interactions that may be playing an important role in this context.

## RESULTS

### Data exploration

**Figure 1:**
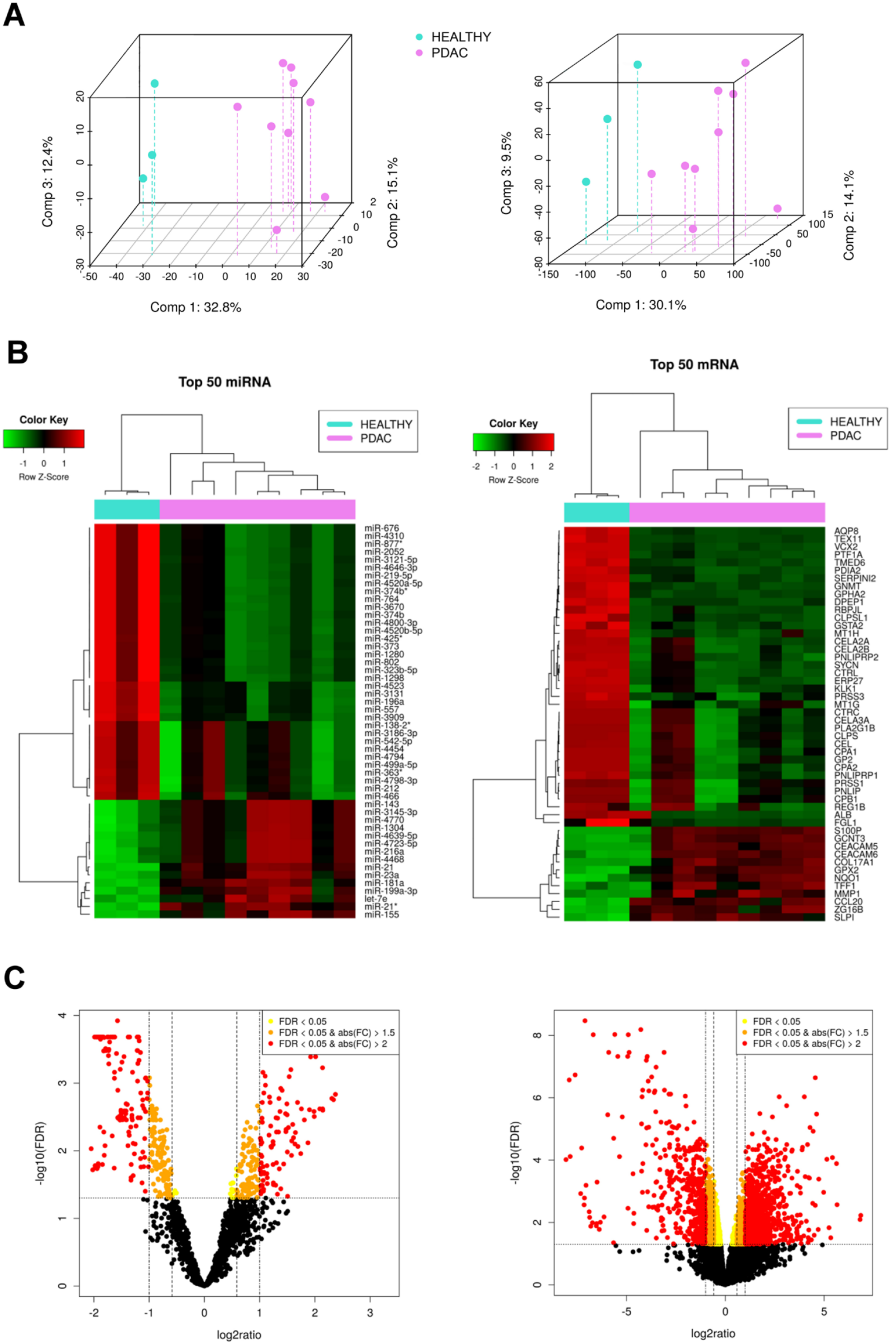
Exploratory analysis of paired miRNA and mRNA expression in pancreatic cancer samples **(A)** 3d-Principal Components Analysis plots, based on correlation matrix, for miRNA (left) and mRNA (right) expression in Healthy (n=3) amd PDAC (n=9) tissue samples. **(B)** Heatmaps of the top 50 most differentially expressed miRNAs and mRNAs, respectively, sorted by absolute FC (all of them having FDR<0.05). **(C)** Volcano plot of the miRNAs (left) and mRNAs (right) highlighting in yellow those with FDR < 0.05, orange FDR < 0.05 and absolute FC > 1.5, and red FDR < 0.05 and absolute FC > 2.

The dataset consists on 3 controls (healthy pancreatic tissue samples) and 9 cases (PDAC tissue samples) with paired miRNA-mRNA data, including the expression of 1733 miRNAs and 18570 mRNAs. Supplementary Table 1 shows clinical information related to these patients. Figure 1A shows Principal Components Analysis of the dataset. We can see that PDAC samples are clearly different from healthy ones depending either on miRNA or on mRNA profiling.

### Top differentially expressed miRNAs or mRNAs

Figure 1B shows the most differentially expressed miRNAs and mRNAs between PDAC and healthy tissue. There are 201 significantly upregulated and 342 significantly downregulated miRNAs in our pancreatic cancer set. They represent 31.1% of the total expressed miRNAs. 30 of the these upregulated miRNAs were validated by RT-qPCR in two larger cohorts of pancreatic cancer patients in our previous article [8]. There also are 1613 significantly upregulated and 2030 significantly downregulated mRNAs between PDAC and healthy tissues, representing 19.6% of the total expressed mRNAs. Figure 1C shows their respective volcano plots colouring the miRNAs and mRNAs according to their fold-change (FC). The miRNAs and mRNAs that were selected for further exploration were those with FDR < 0.05 regardless of their FC (highlighted in yellow, orange and red).

### Intersection with miRNA target prediction databases

We then selected the 543 and 3643 significantly deregulated miRNAs and mRNAs, respectively, and computed all possible correlations. Multiple testing corrections with FDR were applied. Among 1978149 possible miRNA-mRNA combinations, there were 959775 that correlated negatively and, among them, we found 443100 miRNA-mRNA pairs where this correlation had FDR < 0.05. This number represented 22.4% of the total miRNA-mRNA possible combinations. Furthermore, we used the information given by 3 miRNA target prediction databases (TargetScan, miRDB, miRSVR) to intersect with the above mentioned correlations.

**Figure 2:**
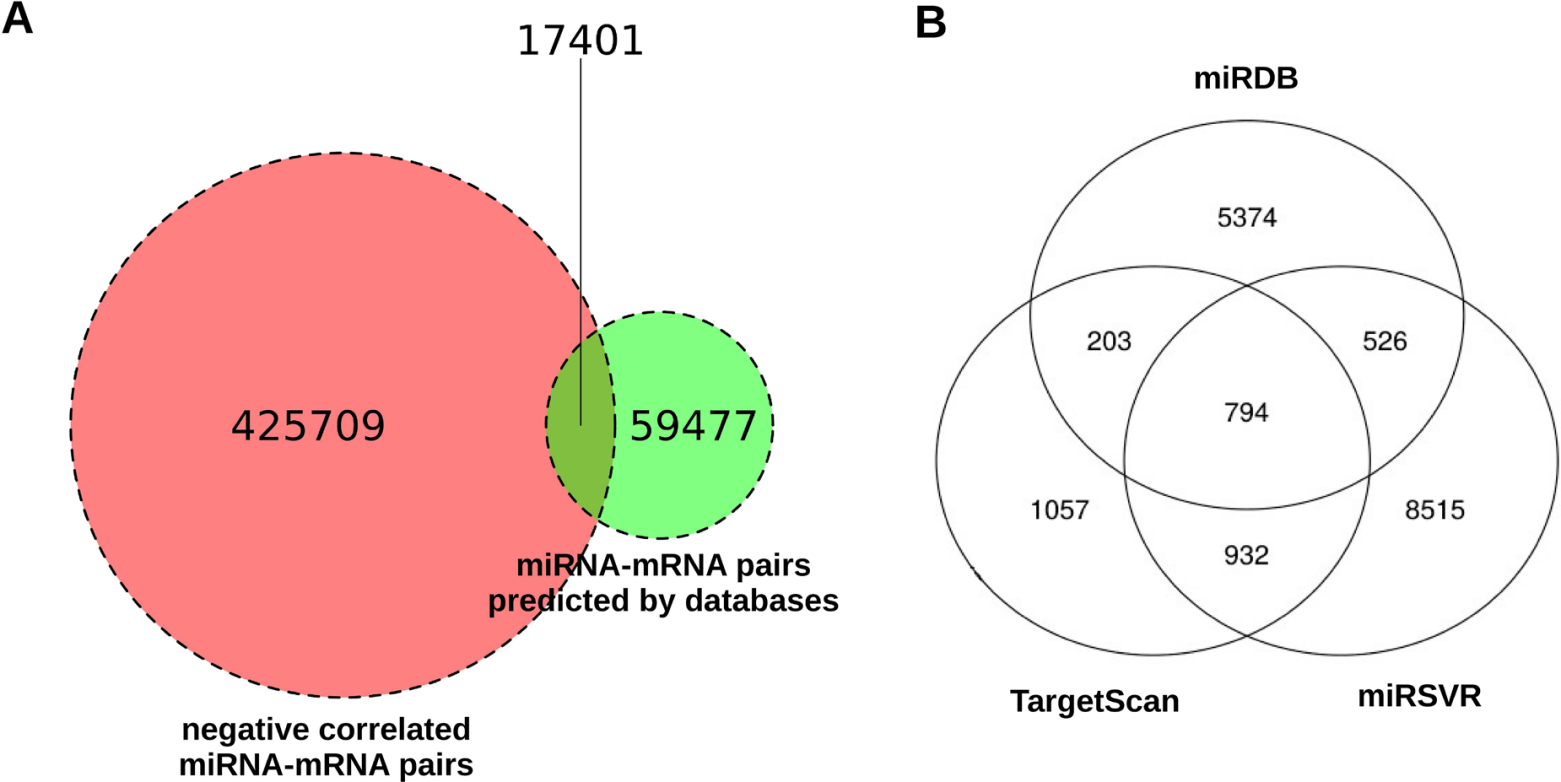
Venn diagrams about the number of miRNA-mRNA interactions predicted by miRComb in the pancreatic cancer context **(A)** Negatively correlated miRNA-mRNA pairs (FDR < 0.05, left), pairs predicted by at least one database (TargetScan, miRDB or miRSVR, right), and miRNA-mRNA pairs that fulfill both conditions. **(B)** Venn diagram showing the overlap between databases among the 17401 miRNA-mRNA pairs that are negatively correlated (FDR < 0.05) and predicted in at least one database (TargetScan, miRDB or miRSVR).

If we only took into account the 3 miRNA target prediction databases, we would have found a total number of 76878 potential miRNA targets present in at least one of them, and using the interaction calculated by miRComb we reduced this number nearly five times, as we found 17401 miRNA-mRNA pairs that were also negatively correlated in our expression data. That means that only 22.6% of the miRNA-mRNA interactions appearing in these databases were found as negatively correlated in our dataset. Figure 2A shows the number of negatively correlated miRNA-mRNA pairs, the number of predicted miRNA-mRNA pairs, and the pairs that fulfill both conditions. Figure 2B shows how many miRNA-mRNA interactions are predicted by each database among the 17401. We can see that miRSVR provided more of the miRComb predicted miRNA-mRNA pairs than the other databases (10767 in total, while TargetScan and miRDB predicted 2986 and 6897 respectively), probably due to the fact that this database has globally more miRNA-mRNA interactions described than the others.

Only 794 of the negatively correlated miRNA-mRNA pairs were simultaneously present in the 3 databases, confirming the little overlap that exists between them. That number corresponds to a 0.88% of the total miRNA-mRNA possible combinations existing from the tissue expression analysis. That means that this step considerably reduces the number of miRNA target interactions that are likely to occur in human pancreatic oncogenesis. Moreover, due to the fact that they have been predicted in the three databases, these interactions could be prioritized ahead of the others in terms of confidence.

**Figure 3:**
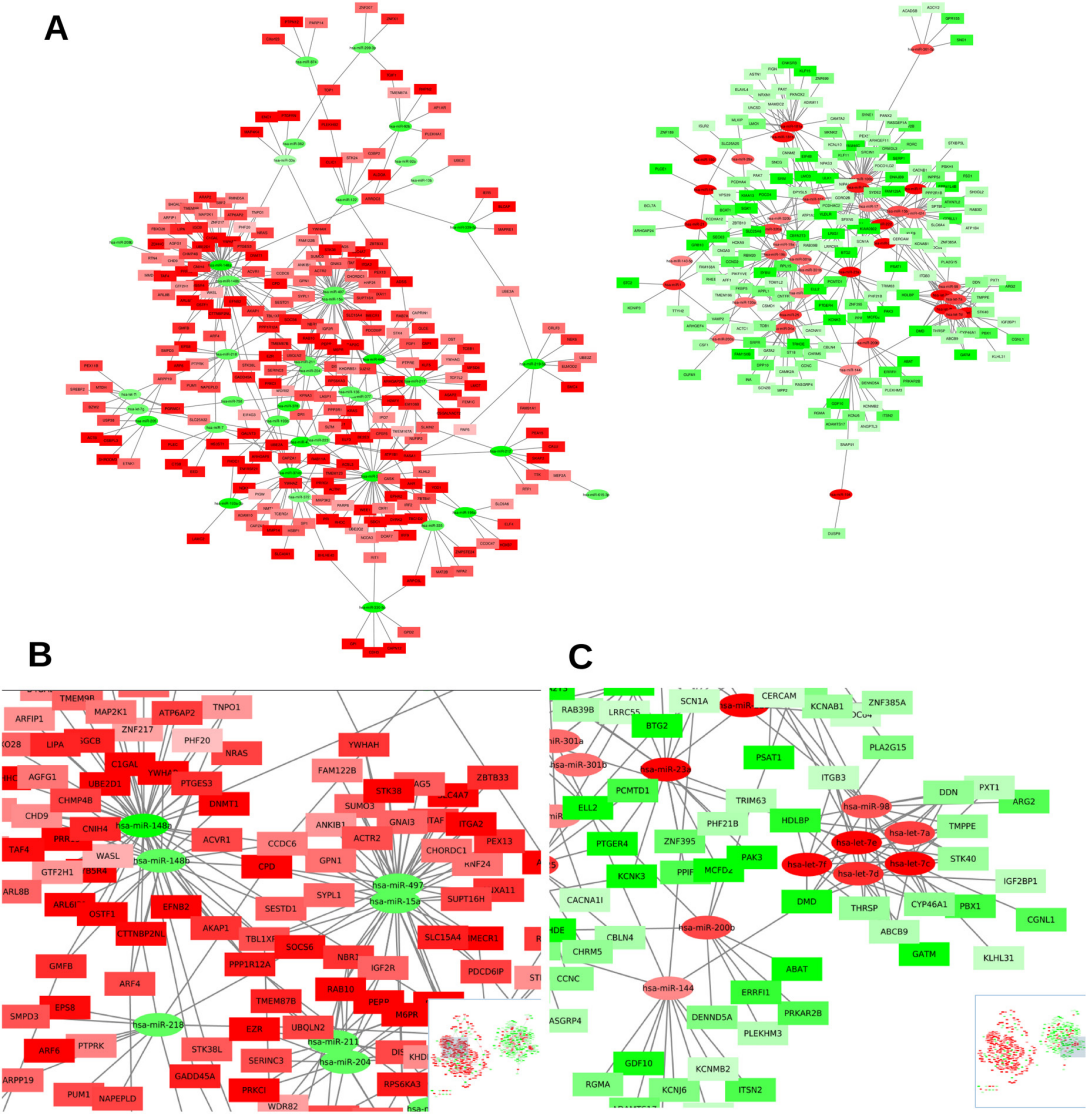
Network of high-confident occurring miRNA-mRNA interactions in pancreatic cancer **(A)** Network of the 794 high-confident miRNA-mRNA interactions occurring in our pancreatic cancer dataset (negatively correlated -FDR < 0.05- and predicted simultaneously in the three used databases: TargetScan, miRSVR and miRDB). Circles represent miRNAs and squares mRNAs, red fill means upregulated miRNA or mRNA, while green fill means downregulated miRNA or mRNA (color intensity is proportional to the FC), lines indicate miRNA-mRNA miRComb interactions. **(B)** Zoom of a left A plot region highlighting the mRNA interactions found for miR-148 family. **(C)** Zoom of a right A plot region highlighting the mRNA interactions found for let-7 family.

Figure 3A shows the network of all these 794 high-confident interactions. MiRNA-mRNA interactions are divided into downregulated miRNAs and their upregulated target mRNAs (left), and upregulated miRNA with downregulated target mRNAs (right). Interestingly, we can see that miRNAs from the same family share most of their target mRNAs, which is why they are represented close to each other. For example, miR-148a and miR-148b are members of the miR-148 family and appear close to each other on the left part of the network (Figure 3A and 3B). That means they share most of their targets, as it can be observed. Similarly, miR-15a and miR-497 share most of their targets and they belong to the same miR-15 family (Figure 3B). We can also observe, in the right part of Figure 3A, that members of the let-7 family (let-7a/c/d/e/f and miR-98) are clustered together according to their miRComb predicted miRNA-mRNA interactions (Figure 3C). In the same way, miR-181a and miR-181b, members of the same miR-181 family, appear together sharing most of their targets. The same occurs for miR-93 and miR-106b that belong to the same family, for miR-320a and miR-320b and for miR-19a and miR-19b (right part Figure 3A) that are clustered together, respectively, according to their miRComb targets.

### MiRComb results in the pancreatic cancer set

**Figure 4:**
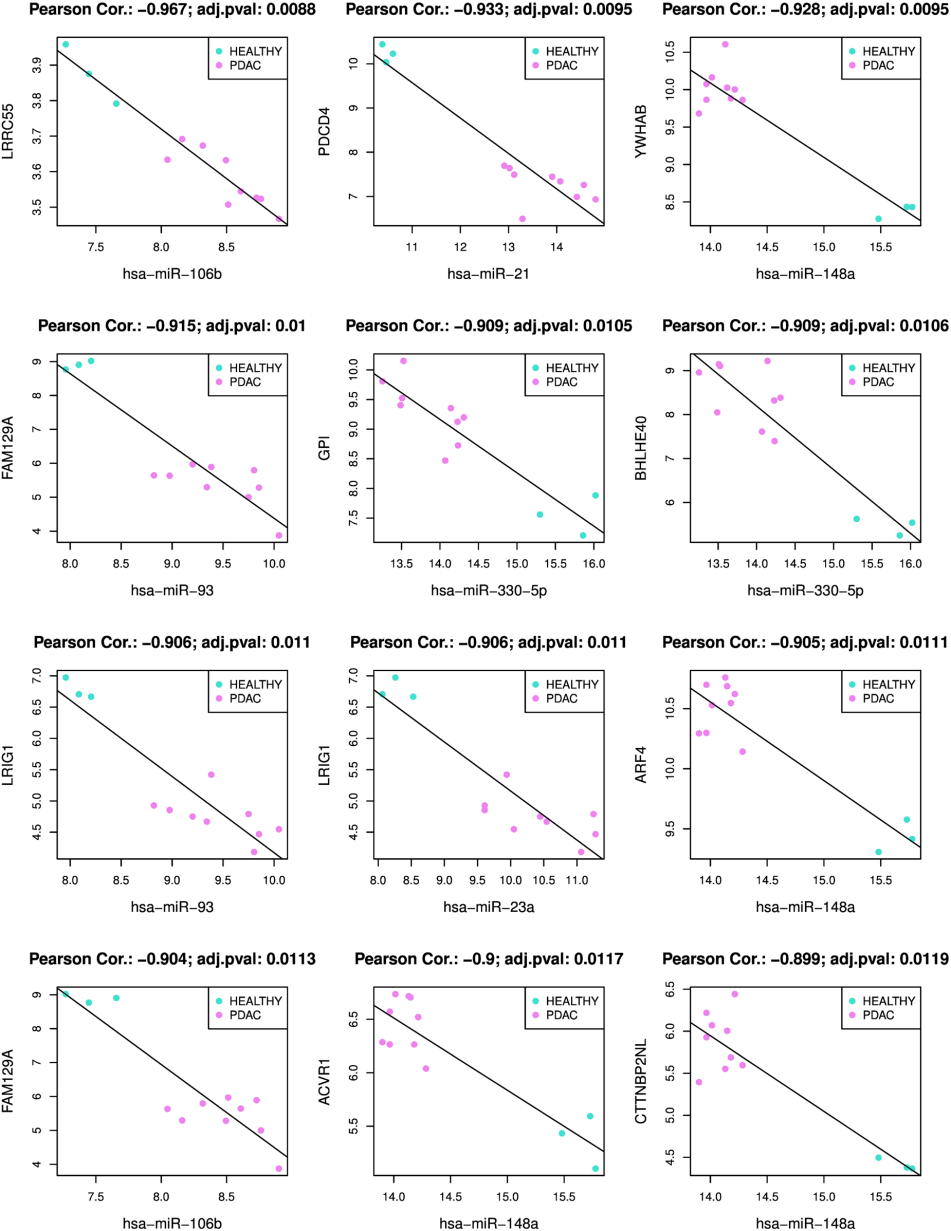
Plot of the top 12 miRNA-mRNA miRComb interactions occurring in pancreatic cancer All miRNA-mRNA pairs are negatively correlated, sorted by correlation FDR, FDR < 0.05 and predicted simultaneously in the three used databases: TargetScan, miRSVR and miRDB.

Supplementary Table 2 shows all 17401 significant miRNA-mRNA interactions found by MiRComb in our pancreatic cancer set of samples. The most significant miRNA-mRNA interactions are detailed below. Table 1 shows the top 50 miRNA-mRNA pairs with most significant negative correlations obtained from miRComb, that appear simultaneously in the three mentioned databases. These would be the miRNA-mRNA interactions that are more likely to occur in a pancreatic cancer context. Figure 4 shows miRNA-mRNA expression correlation for the first 12 most significant miRNA-mRNA pairs of that table. These miRNA-mRNA interactions are miR-106b-LRRC55, miR-21-PDCD4, miR-148a-YWHAB, miR-93-FAM129A, miR-330-5p-GPI, miR-330-5p-BHLHE40, miR-93-LRIG1, miR-23a-LRIG1, miR-148a-ARF4, miR-106b-FAM129A, miR-148a-ACVR1, miR-148a-CTTNBP2NL. PDCD4, GPI, and BHLHE4 are proteins with a described role in pancreatic cancer and, consequently, information about factors that can modulate their expression is important. Concerning the other targets that have not been yet related to pancreatic cancer, this information provides knowledge about new potential pathways playing a role in PDAC. Interestingly, among the miRNAs participating in the 50 most significant miRNA-mRNA interactions we can find: miR-106b, miR-93, miR-148a, miR-330-5p that could be interacting with more than 4 different targets at the same time.

**Table 1:** Top 50 miRNA-mRNA interactions predicted by miRComb

miRNA	mRNA	cor	FDR	FC.miRNA	FC.mRNA	dat.sum
miR-106b	LRRC55	-0,97	0,009	2,07	-1,23	3
miR-21	PDCD4	-0,93	0,010	9,91	-7,90	3
miR-148a	YWHAB	-0,93	0,010	-2,98	3,11	3
miR-93	FAM129A	-0,92	0,010	2,60	-11,48	3
miR-330-5p	GPI	-0,91	0,011	-3,64	3,38	3
miR-330-5p	BHLHE40	-0,91	0,011	-3,64	7,97	3
miR-93	LRIG1	-0,91	0,011	2,60	-4,13	3
miR-23a	LRIG1	-0,91	0,011	4,40	-4,13	3
miR-148a	ARF4	-0,91	0,011	-2,98	2,11	3
miR-106b	FAM129A	-0,90	0,011	2,07	-11,48	3
miR-148a	ACVR1	-0,90	0,012	-2,98	2,11	3
miR-148a	CTTNBP2NL	-0,90	0,012	-2,98	2,76	3
miR-107	PDK4	-0,90	0,012	2,08	-12,85	3
miR-106b	LMO3	-0,89	0,012	2,07	-4,07	3
miR-148a	C1GALT1	-0,89	0,012	-2,98	6,38	3
miR-330-5p	CAPN12	-0,89	0,012	-3,64	3,99	3
miR-148a	TBL1XR1	-0,89	0,013	-2,98	2,06	3
miR-320b	KIAA1324	-0,89	0,013	1,66	-12,22	3
miR-320a	LMO3	-0,88	0,013	2,14	-4,07	3
miR-93	SCN1A	-0,88	0,014	2,60	-1,25	3
miR-148a	CNIH4	-0,87	0,014	-2,98	2,46	3
miR-148a	DNMT1	-0,87	0,014	-2,98	3,09	3
miR-320b	RPL15	-0,87	0,014	1,66	-2,09	3
miR-193b	TNFRSF21	-0,87	0,014	-2,05	8,10	3
miR-148a	UBE2D1	-0,87	0,014	-2,98	3,74	3
miR-181a	LMO3	-0,87	0,014	5,17	-4,07	3
miR-193b	YWHAZ	-0,87	0,014	-2,05	2,59	3
miR-424	LRIG1	-0,86	0,014	1,82	-4,13	3
miR-106b	PDCD1LG2	-0,86	0,014	2,07	-1,30	3
miR-130a	LRIG1	-0,86	0,015	1,74	-4,13	3
miR-497	ITGA2	-0,86	0,015	-1,94	23,44	3
miR-15a	ITGA2	-0,86	0,015	-1,96	23,44	3
miR-34a	VAMP2	-0,86	0,015	2,05	-1,50	3
miR-155	SCN1A	-0,86	0,015	4,03	-1,25	3
miR-299-3p	TOP1	-0,86	0,015	-1,87	2,35	3
miR-367	TOB1	-0,86	0,015	1,61	-1,60	3
miR-330-5p	ARPC5L	-0,86	0,015	-3,64	3,17	3
miR-19b	RBM20	-0,86	0,015	2,00	-1,80	3
miR-34a	INA	-0,86	0,015	2,05	-1,72	3
miR-148a	CPD	-0,86	0,015	-2,98	3,44	3
miR-148a	GMFB	-0,86	0,015	-2,98	2,37	3
miR-374b	NMT1	-0,86	0,015	-3,79	1,71	3
miR-373	RAB11A	-0,86	0,015	-3,76	3,29	3
miR-374b	TCERG1	-0,85	0,015	-3,79	1,59	3
miR-373	CAPZA1	-0,85	0,015	-3,76	2,30	3
miR-373	PFKP	-0,85	0,015	-3,76	14,95	3
miR-144	ANGPTL3	-0,85	0,015	1,63	-1,27	3
miR-19b	SLC25A6	-0,85	0,015	2,00	-3,36	3
miR-93	PDCD1LG2	-0,85	0,015	2,60	-1,30	3
miR-148a	EFNB2	-0,85	0,015	-2,98	3,80	3

MiRNA-mRNA interactions are sorted by FDR and are predicted simultaneously in the three used databases (TargetScan, miRSVR and miRDB; dat.sum=3).

Table 2 shows the top 10 miRNAs according to its number of targets predicted by miRComb, appearing in at least 1 of the used databases. We have added a column showing the number of potential targets initially predicted by the pre-existing target prediction databases, indicating the reduction of the number of miRNA-mRNA interactions provided by miRComb. Each miRNA show different degrees of reduction, being 77% the global average percentage of reduction. It is worth noting that these 10 miRNAs together (miR-374b, miR-148a, miR-181a, miR-373, miR-320a, miR-448, miR-93, miR-106b, miR-217, miR-539) could potentially be regulating 41% of the mRNAs significantly altered in PDAC. Conversely, Table 3 shows the top 10 mRNAs with more miRNAs targeting them.

**Table 2:** Top 10 miRNAs by number of targets

miRNA	#tgts	Orig	Cum %	Names of mRNA targets (Top 20)
*miR-374b*	381	(866, 56%)	10,46%	PMEPA1, CD58, TMSB10, CCL20, CTSB, HSPH1, DNMT1, DIS3, ELF1, UBAC2, FAT1, CCDC47, PTPN12, COPB1, FAM122B, IL8, CTTNBP2NL, FAM96A, H2AFY, ACVR1
*miR-148a*	363	(595, 39%)	16,85%	HLA-A, KLF5, CTSB, TNFRSF21, TMSB10, BID, TMEM123, KCNK1, B2M, PGRMC1, YWHAB, TAGLN2, ENDOD1, PTPN12, UBE2A, ACSL3, MYO1D, AMMECR1, PLEKHB2, ACTG1
**miR-181a**	259	(828, 69%)	23,96%	PDCD4, IFRD1, DFFB, EPB41L4B, ANGPT1, LRIG1, KCNN1, NUCB2, DMGDH, FKBP11, EPB41, TMED6, LMO3, VCX2, MYO15A, RPL15, SLC25A53, PSAT1, ITSN2, SPATA20
*miR-373*	258	(647, 60%)	26,52%	HLA-A, ENDOD1, B2M, PGRMC1, BID, DIS3, TAGLN2, CCDC47, PTPN12, MDK, PON2, MYO1D, SKAP2, CTTNBP2NL, FAM96A, IL8, H2AFY, PSMA2, ACVR1, C1D
**miR-320a**	252	(751, 66%)	31,62%	WNT9B, PDCD4, TMED6, PAIP2B, SFTPC, ADRA1B, MS4A10, HHIPL1, CACNB1, AOX1, IFRD1, SND1, CECR2, GPHA2, KCNAB1, OSBP2, ERO1LB, EPB41L4B, LMO3, BACE1
*miR-448*	245	(608, 60%)	33,24%	ENDOD1, GBP2, LITAF, LIMS1, DNMT1, ELF1, PTPN12, IL8, FAM96A, VPS13C, SEPT10, SKAP2, CTTNBP2NL, FAM122B, CALM2, RBM41, PPFIA1, IVNS1ABP, NEK6, PFKP
**miR-93**	238	(813, 71%)	36,62%	IFRD1, FAM129A, LRIG1, ATXN7L2, MLC1, EPB41L4B, SH2D5, ANGPT1, ISM2, MS4A10, SYBU, SCN1A, MYO15A, PCMTD1, FBXO24, SLC46A2, EPB41, ITSN2, PAIP2B, WNT9B
**miR-106b**	234	(766, 69%)	37,47%	LRRC55, FNDC5, ZNF385A, SH2D5, FAM129A, MYT1, MLC1, LMO3, IFRD1, C17orf67, KPNA7, APOBEC3H, SLC41A1, TIMM8A, ATOH8, PAIP2B, ARHGAP18, ERO1LB, PRND, MUM1L1
*miR-217*	230	(533, 57%)	39,28%	TNFRSF21, CTNNA1, ARPC2, CLINT1, RAB11A, YWHAH, KLF5, PFKP, MAP4K4, YWHAB, CAP1, PTTG1IP, RAC1, SPTLC2, ADAM9, PRKCI, ISG20, TES, DDX60, TMEM87B
*miR-539*	225	(868, 74%)	41,23%	CCDC109B, NQO1, SULF2, KCNK1, MARCKSL1, ITGA2, PSMB8, ARPC2, DENND2D, HSBP1, SLC44A1, MRPL50, B2M, ENC1, FAM108C1, MAT2B, GCC2, HLA-A, DYNLT1, PNP

Top 10 miRNA with more targets (each miRNA-mRNA pair has FDR < 0.05 and appears at least 1 times in the following databases: TargetSan, miRSVR, miRDB). MiRNAs in bold are upregulated in PDAC, miRNAs in italics are downregulated in PDAC. #tgts: Number of target mRNAs; orig.: number of miRNA-mRNA pairs predicted in at least one database (considering positive and negatively correlated miRNA-mRNA pairs) and percentage of these original pairs that are removed after considering only negatively correlated miRNA-mRNA pairs. Cum %: percentage of deregulated mRNAs that are regulated by the miRNAs, cumulatively. Names of the top 20 mRNA targets are sorted by correlation.

**Table 3:** Top 10 mRNAs by number of miRNAs

mRNA	#miRNA tgts	Names of miRNA (Top 20)
**TBL1XR1**	39	miR-148a, miR-148a^*^, miR-4712-3p, miR-3666, miR-217, miR-4668-5p, miR-4429, miR-15a, miR-497, miR-619, miR-377, miR-548l, miR-211, miR-876-5p, miR- 338-3p, miR-148b, miR-548n, miR-548f, miR-548g, miR-4474-3p
**CTTNBP2NL**	36	miR-148a, miR-2052, miR-3167, miR-373, miR-374b, miR-448, miR-330-5p, miR-4463, miR-196a, miR-302c^*^, miR-567, miR-3168, miR-323-3p, miR-891b, miR-193b, miR-372, miR-377, miR-876-5p, miR-122, miR-136
**YWHAZ**	36	miR-193b, miR-217, miR-4429, miR-375, miR-339-5p, miR-636, miR-122, miR-758, miR-4474-3p, miR-92b, miR-204, miR-876-5p, miR-136, miR-548am, miR-211, miR-802, miR-641, miR-448, miR-7, miR-373
**AMMECR1**	33	miR-148a, miR-196a, miR-4310, miR-4253, miR-4700- 5p, miR-448, miR-618, miR-4436b-5p, miR-1236, miR- 4428, miR-4668-5p, miR-497, miR-15a, miR-876-5p, miR-148b, miR-548g, miR-548n, miR-4679, miR-548am, miR-548m
**TNPO1**	33	miR-299-3p, miR-154, miR-211, miR-4668-5p, miR- 325, miR-4418, miR-218, miR-548n, miR-208b, miR- 148a, miR-4469, miR-15a, miR-548f, miR-548g, miR- 497, miR-4504, miR-548m, miR-548h, miR-548am, miR-4775
**CCDC6**	32	miR-302c^*^, miR-567, miR-148a, miR-30a^*^, miR-4725- 3p, miR-148a^*^, miR-3666, miR-641, miR-4310, miR-373, miR-802, miR-374b^*^, miR-3685, miR-875-5p, miR-330-5p, miR-557, miR-497, miR-15a, miR-122, miR-211
**CPD**	32	miR-148a, miR-30a^*^, miR-635, miR-373, miR-641, miR-196a, miR-448, miR-497, miR-15a, miR-211, miR-377, miR-4255, miR-378e, miR-548f, miR-876-5p, miR-148b, miR-548am, miR-204, miR-338-3p, miR-4477b
**CPSF6**	32	miR-148a, miR-4761-3p, miR-4741, miR-497, miR- 15a, miR-802, miR-377, miR-204, miR-548f, miR- 548am, miR-548n, miR-670, miR-136, miR-4762-3p, miR-548m, miR-448, miR-548h, miR-618, miR-4775, miR-2355-3p
**G3BP2**	32	miR-148a, miR-374b, miR-802, miR-4253, miR-448, miR-219-5p, miR-4463, miR-217, miR-4688, miR-3184, miR-148a^*^, miR-323-3p, miR-485-3p, miR-497, miR- 4668-5p, miR-15a, miR-122, miR-212, miR-335, miR- 148b
**PDCD6IP**	32	miR-148a, miR-217, miR-26b^*^, miR-3144-3p, miR- 323-3p, miR-548l, miR-148b, miR-211, miR-15a, miR- 497, miR-876-5p, miR-4477b, miR-548g, miR-204, miR-875-5p, miR-3140-5p, miR-4262, miR-4775, let-7i, miR-485-3p

Top 10 mRNA with more miRNAs targeting them (each miRNA-mRNA pair has FDR < 0.05 and appears at least 1 times in the following databases: TargetScan, miRSVR, miRDB). mRNAs in bold are upregulated in PDAC. Names of the top 20 miRNA targeting the mRNA are sorted by correlation.

**Figure 5:**
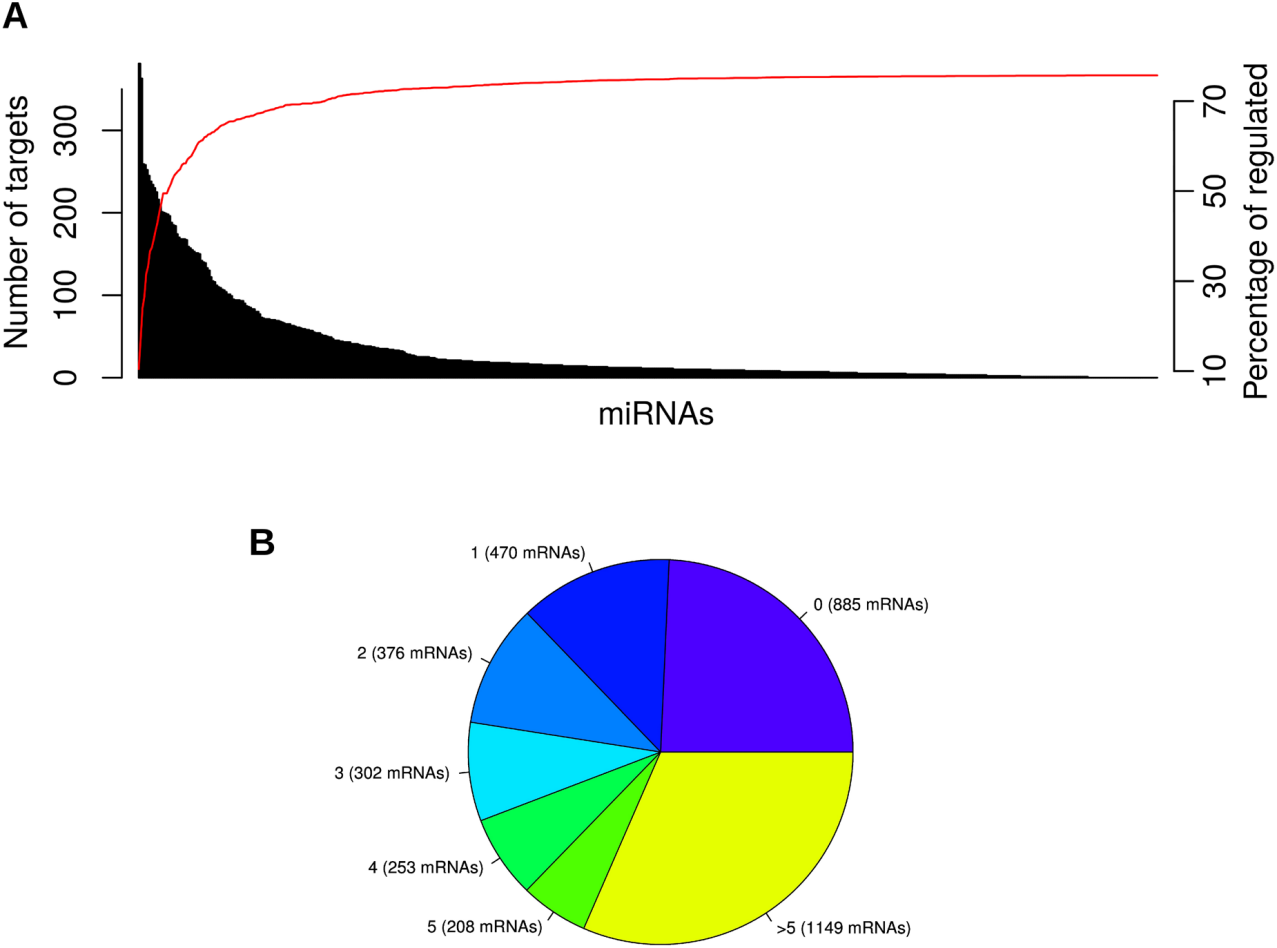
Barplot an piechart summarizing the number of miRComb interactions per miRNA and mRNA MiRNA-mRNA miRComb interactions are those negatively correlated (FDR < 0.05) and predicted in at least one database (TargetScan, miRVR or miRDB). **(A)** Barplot showing the number of mRNA targets per each miRNA (each bar represents a miRNA and they are sorted by number of targets). Red line means the percentage of mRNAs that are cumulatively regulated by the previous miRNAs. **(B)** Pie chart representing the number of miRNAs that are regulating each mRNA.

As an overview, Figure 5A shows the number of mRNA targets per each miRNA and the cumulative number of mRNAs that are being regulated by the previous miRNAs. Interestingly, 50% of the significantly deregulated mRNAs are regulated by the top 17 miRNAs, and almost no mRNAs are added by the last ones. Moreover, Figure 5B shows the number of mRNAs targeted by 0, 1 or more miRNAs. It is important to point out that 1149 mRNAs (representing 41.7% of the significantly deregulated ones in PDAC samples) are targeted by more than 5 miRNAs. Furthermore, both figures show that 75% of the significantly deregulated mRNAs are targeted by at least one miRNA.

**Figure 6:**
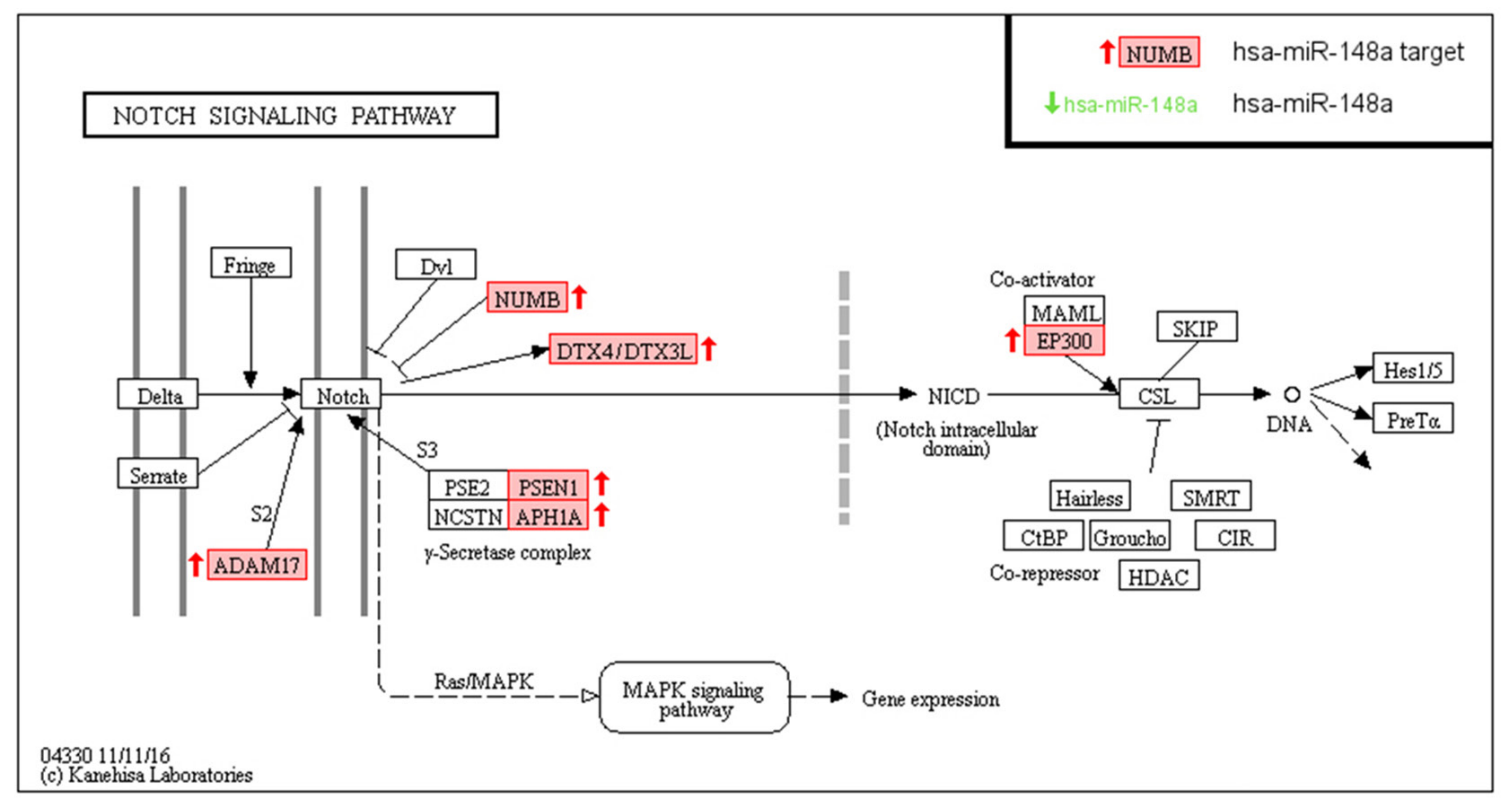
MiR-148a targets involved in Notch signaling pathway from KEGG analysis, in the context of pancreatic cancer miR-148a miRComb targets (mRNAs that are negatively correlated with miR-148a -FDR < 0.05- and predicted in at least one database of TargetScan, miRVR or miRDB) are highlighted in red.

It is interesting to highlight that miR-148a also appears on the top list from Table 2, emphasizing its importance in pancreatic carcinogenesis. Target enrichment analysis of these miR-148a targets by KEGG revealed significant enrichment only in the Notch Signaling Pathway (FDR<0.02). Consistently, target enrichment analysis with other methods as GO-Biological Processes or Reactome also showed significant enrichment in this Notch pathway among others. Supplementary Table 3 shows all the results obtained from the three different target enrichment analysis performed. Figure 6 shows as 7 key members (NUMB, DTX4, DTX3L, PSEN1, APH1A, ADAM17 and EP300) of that pathway are predicted as miRComb miR-148a targets in our pancreatic cancer samples.

### Assessment of miR-148a targets from Notch pathway in a pancreatic cancer cellular model

**Figure 7:**
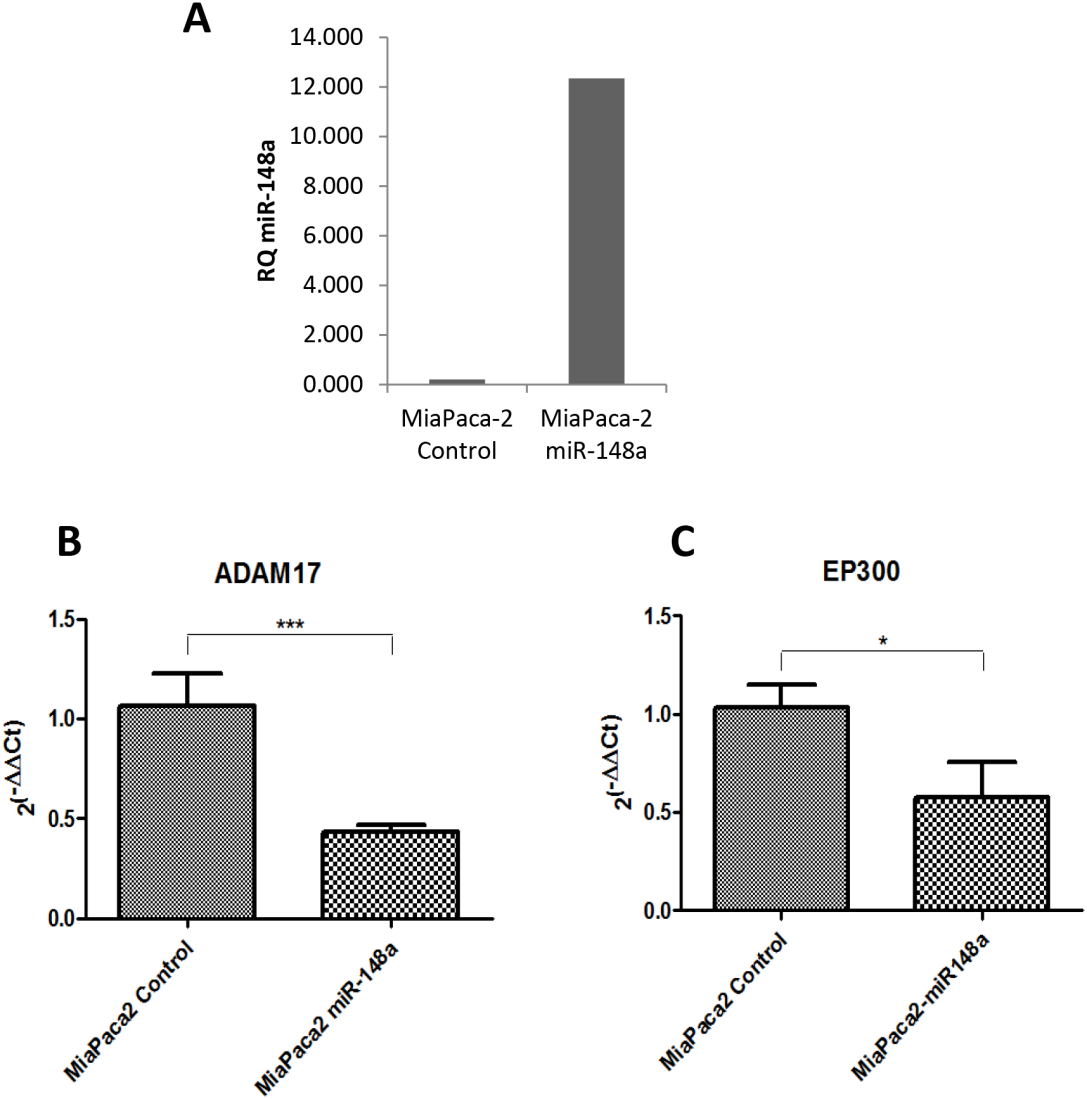
Evaluation of miR-148a targets in a pancreatic cancer cellular model overexpressing miR-148a **(A)** qRT-PCR basal expression of miR-148a in the MiaPaca-2 overexpressing miR-148a clone (MiaPaca-2 miR-148a) and MiaPaca-2 scrambled miRNA transfected (MiaPaca-2 Control) **(B)** qRT-PCR expression of ADAM17 in both MiaPaca-2 Control and MiaPaca-2 miR-148a cells (n=6). **(C)** qRT-PCR expression of EP300 in both MiaPaca-2 Control and MiaPaca-2 miR-148a cells (n=6). Relative expression of mRNAs was calculated as 2^(-∆∆Ct)^. ^*^P<0.05, ^***^P<0.001.

In order to check those proposed miR-148a targets in the context of pancreatic cancer we took advantage of the pancreatic cancer cellular model (MiaPaCa-2) stably overexpressing miR-148a, that we have previously generated [11]. We measured the expression of key members of the Notch Signaling Pathway (NUMB, DTX4, DTX3L, PSEN1, APH1A, ADAM17 and EP300) in the MiaPaCa-2-miR-148a by qRT-PCR, and compared it with the basal levels of the control pancreatic cancer cell line MiaPaCa-2, expressing very low levels of miR-148a (Figure 7A). Among the seven targets analyzed, ADAM17 and EP300, showed significantly decreased expression in the presence of high levels of miR-148a compared to the low miR-148a levels expressed by the control cell line (Figure 7B and 7C). These results show that the expression of Notch Signaling components ADAM17 and EP300 is, at least in part, regulated by miR-148a in a pancreatic cancer context.

### Assessment of miR-21 targets in a pancreatic cancer cellular model

**Figure 8:**
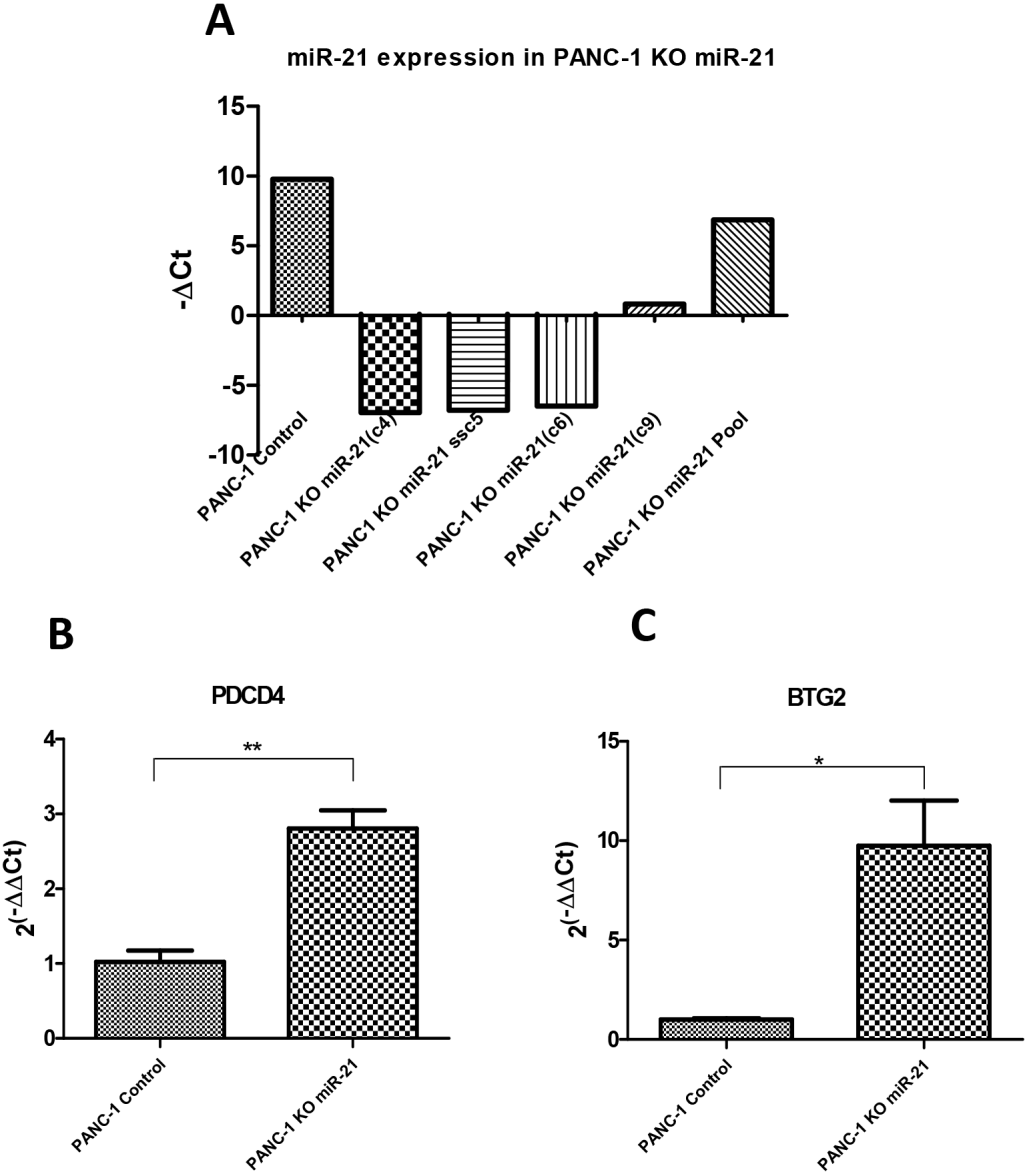
Evaluation of miR-21 targets in a CRISPR/Cas9 generated miR-21 deficient pancreatic cancer cellular model **(A)** qRT-PCR expression of miR-21 in the PANC-1 CRISPR/Cas9 generated miR-21 knock-out (KO) clones and PANC-1 Control. **(B)** qRT-PCR expression of PDCD4 in both PANC-1 Control and PANC-1 KO miR-21 cells (n=3). **(C)** qRT-PCR expression of BTG2 in both PANC-1 Control and PANC-1 KO miR-21 cells. Relative expression of mRNAs was calculated as 2^(-∆∆Ct)^. ^*^P<0.05, ^**^P<0.01.

To go one step further, we focused on miR-21 because it is one of the most up-regulated miRNAs in PDAC as we can see in Table 1 with a FC=9.91. This wide range of expression makes it a good candidate to test some of its mRNA targets. In Table 4 is shown the list of top miRComb predicted targets for miR-21 (present in more than 1 database). In order to experimentally evaluate some of these miR-21 predicted targets in the context of pancreatic cancer, we selected PDCD4 and BTG2 from that list for being also highly down-regulated in PDAC (FC=-7.88 and FC=-5.53, respectively). We generated a pancreatic cancer cellular model (PANC-1) lacking miR-21 expression by using CRISPR/Cas9 methodology. After confirming which clones did not express miR-21 (Figure 8A), we measured the basal expression of PDCD4 and BTG2 in three PANC-1 KO miR-21 clones (c4, c5 and c6), and compared it with the control pancreatic cancer cell line PANC-1 expressing high levels of miR-21. As expected, both, PDC4 and BTG2, showed significantly increased expression in the absence of miR-21 compared to the control miR-21 expressing cell line (Figure 8B, 8C). These results show that the expression of these genes is, at least in part, regulated by miR-21 in a pancreatic cancer context.

**Table 4:** MiR-21 miRComb targets

miRNA	mRNA	cor	FDR	TargetScan	miRSVR	miRDB	dat.sum
miR-21	PDCD4	-0,93	0,010	1	1	1	3
miR-21	PAIP2B	-0,90	0,012	1	1	0	2
miR-21	SMARCD1	-0,88	0,013	1	1	0	2
miR-21	SERP1	-0,85	0,015	1	1	0	2
miR-21	B3GAT2	-0,84	0,016	0	1	1	2
miR-21	BTG2	-0,84	0,016	1	1	0	2
miR-21	BCL7A	-0,83	0,017	1	1	1	3
miR-21	ALX4	-0,83	0,017	1	0	1	2
miR-21	SEC63	-0,81	0,019	0	1	1	2
miR-21	RNF182	-0,79	0,021	0	1	1	2
miR-21	ARHGAP24	-0,79	0,021	1	1	1	3
miR-21	STK40	-0,79	0,022	1	0	1	2
miR-21	CNTFR	-0,78	0,023	1	1	0	2
miR-21	NPAS3	-0,77	0,024	1	1	0	2
miR-21	ABAT	-0,77	0,025	0	1	1	2
miR-21	KLF9	-0,76	0,026	1	1	0	2
miR-21	EPM2A	-0,74	0,028	0	1	1	2
miR-21	ADCY2	-0,73	0,030	0	1	1	2
miR-21	PIKFYVE	-0,70	0,036	1	1	1	3
miR-21	SLC16A10	-0,70	0,037	1	1	0	2

Only significant targets with negative correlation (FDR < 0.05) and present in 2 or 3 of the databases (TargetScan, miRSVR or miRDB) are shown.

## DISCUSSION

In this study we have shown that the final miRComb number of miRNA-mRNA interactions in our set of pancreatic cancer samples is 17401, that corresponds to a 22.6% of the 76878 potential miRNA targets predicted by at least one of the miRNA target prediction databases used in this study (TargetScan, miRSVR, miRDB). The expression of these 17401 miRNA-mRNA pairs is negatively correlated (FDR < 0.05) and predicted in at least one of the above mentioned databases. Although the experimental confirmation of all these interactions have not been done, and therefore there may be some false positives among them, this analysis considerably filters the number of potential miRNA target interactions and help to focus more directly on those that are more likely to occur in pancreatic carcinogenesis.

The little overlap found on the predictions of the three mentioned databases reinforces the idea that is better to use more than one database and take advantage of other sources of information such as miRNA and mRNA expression to filter out the results. Concordantly, previous studies suggest that combinations of miRNA-mRNA databases have less false positives [12]. In our study, we used the number of coincidences across databases to prioritize those miRNA-mRNA interactions that would have a more important role in pancreatic carcinogenesis.

Specifically, miRNA-mRNA interactions shown in Table 1 should be those more likely to occur in a pancreatic cancer context, given that they are the most negative correlated that appear simultaneously in the three target prediction databases. Among them, it is noteworthy that there are miRNAs that have been previously described as important in pancreatic cancer for being significantly up-regulated or down-regulated in tumor tissue in comparison to healthy pancreas. For example, miR-106b, miR-107, miR-130a, miR-34 [9], miR-93, miR-155, miR-181a, miR-21, miR-23a, miR-320a [8], miR-193b, miR-320b [13] are significantly up-regulated and miR-148a [11, 14], miR-330-5p [15], miR-373 [16] significantly down-regulated. It is important to highlight the high number of miR-148a interactions that appear among the most significant (12/50), suggesting it may have a central role in pancreatic tumorigenesis. It is likely that miR-148a is involved in more pancreatic cancer pathways than those reported so far for apoptosis and cell survival [17, 18]. In that sense, functional enrichment analysis according to miR-148a targets by KEGG, Reactome and GO revealed significant target enrichment in the Notch signaling pathway, among others. Figure 6 shows proteins involved in that Notch pathway highlighting those that appeared as miRComb predicted targets for miR-148a as NUMB, DTX4, DTX3L, PSEN1, APH1A, ADAM17 and EP300. In order to experimentally evaluate these predicted interactions, we analyzed the expression of these targets in a pancreatic cancer cell model overexpressing miR-148a in a stable way. Two components of the Notch signaling pathway, ADAM17 and EP300, could be confirmed as miR-148a targets in that cellular model. In recent years, accumulated evidence has demonstrated that Notch signaling pathway plays critical roles in the development and progression of PDAC [19]. It has been well documented that the Notch signaling pathway is critical for cell proliferation, differentiation, development and homeostasis [20]. Reactivation of Notch signaling is observed in early PDAC pathogenesis and persists throughout the progression of the disease [21–25]. However, no relationships between miR-148a and Notch signaling pathway have been described so far in pancreatic cancer and more studies would be needed to confirm and explore this relationship. Consistently, evidences about miR-148a regulation of Notch pathway members have been recently reported in hepatocellular carcinoma [26].

Concordantly, miR-148a, together with miR-374b, are the miRNAs with more miRComb predicted targets (363 and 381, respectively) as shown in Table 2. MiRNAs appearing in that table probably are those playing more central roles in PDAC because they are the ones with more targets and they would regulate a huge number of mRNAs simultaneously. Interestingly, most of these miRNAs are coincident with those appearing in Table 1 (miR-374b, miR-148a, miR-181a, miR-373, miR-320a, miR-93, miR-106b, miR-497, miR-23a, miR-19b, miR-107, miR-15a, miR-330-5p, miR-144), indicating that, apart from being targeting many mRNAs, these miRNAs are participating in the most reliable interactions. Furthermore, as we have mentioned above, most of them have already been reported to be significantly deregulated in PDAC. Altogether suggests these miRNAs may constitute central players of pancreatic tumorigenesis and could be new therapeutic target candidates.

Strikingly, the top 10 miRNAs with more targets are able to regulate 41% of the deregulated mRNAs in PDAC (Table 2). Moreover, 75% of the mRNAs are regulated by at least one miRNA, and 31.5% of the mRNAs are regulated by more than 5 miRNAs. We also have to bear in mind that there are mRNAs not regulated by one single miRNA, and that competence and cooperativity between miRNAs have also been described [27, 28]. Altogether, these data confirms that miRNAs are acting as fine-tuning regulators of gene expression in pancreatic cancer as also happens in a wide range of diseases [29–31].

Another important miRNA that seems to play important roles in PDAC is miR-21, as is one of the most deregulated miRNAs in PDAC. MiR-21 is currently one of the best studied miRNAs that plays relevant roles in cancer as it is named as oncomiR-21 [32]. It has also been described to have important roles in pancreatic cancer [8, 33–35]. In order to experimentally evaluate if some of these predicted targets could act as miR-21 targets in the context of pancreatic cancer, we selected 2 targets (PDCD4, BTG2) from the top miR-21 targets list (Table 4). Both PDCD4 and BTG2 are described to play a tumor suppressor role in several cancers and are downregulated in PDAC [36]. PDCD4 is also a known target of miR-21 in several types of cancer (colon cancer [37, 38] or diffuse large B-cell lymphoma [39]), including PDAC [40, 41]. BTG2 has been related to pancreatic cancer [42], and the relation between miR-21 and BTG2 interaction has been observed in other cancers (multiple myeloma [43], liver cancer [44], prostate cancer [45]), but they still have not been directly linked in pancreatic cancer.

In this study we have confirmed the involvement of miR-148a-ADAM17, miR-148a-EP300, miR-21-PDCD4 and miR-21-BTG2 interactions in the pancreatic cancer cell with the help of genetically modified pancreatic cancer cellular models (stable overexpression or CRISPR/Cas9 knock-out, respectively). However, we cannot affirm that all the interactions proposed here really exist because they should be experimentally validated one by one. Nevertheless, the aim of this study was to unveil a list of high confident miRNA-mRNA interactions for pancreatic cancer that can be the seed for a high number of studies aiming to understand more deeply the molecular pathogenesis of PDAC.

## MATERIALS AND METHODS

### Samples

A set of 12 surgical pancreatic tissue samples (9 PDAC and 3 healthy) from Hospital Clínic of Barcelona (Barcelona, Spain) patients were included. The same samples were used for both genome-wide miRNA and mRNA profiling. Sample dissection was performed by experienced pathologists who split tissue samples in two different parts: one for gene expression analysis and the other for diagnostic confirmation. Pancreatic tissues were kept on dry ice at all times during handling, flash frozen in liquid nitrogen and stored at -80°C until RNA isolation. Healthy pancreatic samples correspond to the healthy tissue of patients who underwent surgery for other reasons (i.e., ampulloma or neuroendocrine tumours). None of the patients with PDAC had received chemo or radiotherapy before sample collection.

This study was approved by the Institutional Ethics Committee of Hospital Clínic of Barcelona (March 27, 2008) and written informed consent was obtained from all patients in accordance with the Declaration of Helsinki.

Total RNA including miRNA was isolated from frozen macrodissected tissues using the miRNeasy Mini Kit (Qiagen, Valencia, CA, USA), according to the manufacturer protocol. RNA concentrations and purity were evaluated using NanoDrop 1000 Spectrophotometer (Wilmington, DE, USA) and RNA quality was determined by Bioanalyzer 2100 (Agilent, CA, USA).

### Data obtention and processing

Genome-wide miRNA profiling was done by next generation sequencing (NGS) technology on a Genome Analyzer IIx (Illumina, CA, USA) as described in our previous study [8]. MiRNA counts were found according to Mirdeep2 procedure [46]. Reads were aligned to Human Reference Genome GRCh37, and matched to miRBase v.17 in order to find the count miRNAs [47]. Expression was detected for 1733 miRNAs. For this analysis, normalized counts by DESeq [48] were log2-scaled in order to apply the LIMMA-trend procedure [49, 50] and allow for linear models, as suggested by Law (log-cpm values) [49, 50].

Matched genome-wide mRNA profiling was analyzed by microarray technology with Human Genome U219 Gene Expression Arrays (Affymetrix, Santa Clara, CA, USA) and normalized according to LIMMA procedure [49].

### MiRNA-mRNA interactions

MiRNA-mRNA correlations were computed using miRComb package [10]. Briefly, this package selects differentially expressed miRNAs and mRNAs from the same sample, computes miRNA-mRNA correlations and, then, matches them with pre-existing target prediction databases. The final selected miRNA-mRNA interactions are those that their expression correlates in a negative and significant manner, and appear as predicted in at least one of the following databases (TargetScan [51] http://targetscan.org, miRDB [52] http://mirdb.org/miRDB and miRSVR [53] http://www.microrna.org/microrna/home.do).

KEGG, GO and Reactome enrichment analysis were applied with miRComb R package (which implements the hypergeometric test from GOstats R package). MiR-148a miRComb predicted targets (FDR < 0.05) detected in at least one database were used. Supplementary Table 3 shows all the results obtained.

### Cell culture

Human pancreatic cancer cell lines PANC-1 and MiaPaca-2 were obtained from European Collection of Cell Cultures (ECACC, Wiltshire, UK) and cultured in Dulbecco's modified Eagle's medium (GIBCO, Thermo Fisher Scientific, Waltham, MA, USA) supplemented with 10% fetal bovine serum (GIBCO, Thermo Fisher Scientific) and 1% penicillin/streptomycin (GIBCO, Thermo Fisher Scientific). Cells were incubated at 37°C and 5% CO2 in a humidified chamber.

### MiR-148a overexpression in MiaPaca-2 cells

MiaPaca-2 cells stably overexpressing miR-148a (MiaPaca-2 miR-148a) and MiaPaca-2 scrambled miRNA transfected (MiaPaca-2 Control) were obtained by us as previously described [11].

### CRISPR/Cas9 targeting of miR-21 in PANC-1 cells

#### gRNA design

The gRNA of miR-21 was designed using the “CRISPR design tool” from Feng Zhang Lab (http://crispr.mit.edu/). We chose a PAM sequence in the pre-miR-21 region and selected a 20-bp sequence upstream as the targeting sequence (5’-TCATGGCAACACCAGTCGAT-3’). Oligonucleotides of the indicated sequence were purchased from IDT (Leuven, BE), annealed and cloned into the plentiCRISPRv2 vector following Lentiviral CRISPR Tool box instructions from Zhang Lab deposited to Addgene.

#### Verification of gRNA-mediated genome cleavage

HEK293T cells were transfected with the plentiCRISPRv2 containing miR-21 gRNA by CalPhos mammalian transfection kit (Clontech, Takara Bio Company Inc., Mountain View, CA, USA). Cells were treated with 4 μg/ml puromycin for one week. Next, genomic DNA from transfected and wild-type cells was isolated and submitted to PCR amplification of a 555 bp fragment that encompasses miR-21 region using the following primers: Fwd: 5’- CCACACTCTGTCGTATCTGTG-3’ Rev: 5’- AAGTGCCACCAGACAGAAGG-3’. PCR fragments were subjected to SURVEYOR nuclease assay (Transgenomic) and resolved on 1.5% agarose gel. Mutations were confirmed by DNA sequencing.

#### Generation of miR-21-deleted PANC-1 cells

Lentiviral particles were generated by transfection of vectors plentiCRISPRv2miR-21gRNA or plentiCRISPRv2-Control (for control cells), pVSV-G and pCMVΔ8.91 into HEK293T by CalPhos mammalian transfection kit. At 48h the viral supernatants were collected, filtered and added to PANC-1 cells. Three days after transduction, cells were selected in 8 μg/ml puromycin for one week. Next, limiting dilution was carried out to generate individual clones from PANC-1 infected with miR-21gRNA cells and three weeks later several clones were analyzed for DNA mutation and miR-21 expression.

### RNA extraction and target expression analysis by qRT-PCR

Total RNA was isolated from cell cultures using the miRNeasy Mini Kit (Qiagen, Valencia, CA, USA), according to the manufacturer protocol. The final elution volume was 30μL. RNA concentrations and purity were evaluated using NanoDrop 1000 Spectrophotometer (Wilmington, DE, USA). Gene expression levels of several targets were analyzed by qRT-PCR using TaqMan High Capacity cDNA Reverse Transcription Kit (Applied Biosystems Inc., Foster City, CA, USA). A two-step protocol involves reverse transcription, followed by a real time PCR with TaqMan probes. Briefly, 1μg total RNA was used per reverse transcription reaction performed in final volume of 10μL (5μL RNA, 0,4μL of 100mM dNTPs, 0.5μL of Multiscribe Reverse Transcriptase (50U μL-1), 1μL of 10X RT buffer, 0.5μL of RNase inhibitor (20U μL-1), 1μL 10x RT random primers and 1,6 μL Nuclease-free water) and incubated for: 10 minutes, 25°C; 120 minutes, 37°C; 5 minutes, 85°C; hold at 4°C. The 10μL PCR mixture included 4μL cDNA, 6μL of TaqMan 2X Universal PCR Master Mix with no AmpErase UNG and 0.5μL of TaqMan 20X MicroRNA Assay. PCR reactions were incubated in a 384-well optical plate and run on the Viia7 Real-Time PCR System (Applied Biosystems Inc.) as follows: 95°C for 10 min and 50 cycles of 95°C for 15 sec and 60°C for 1 min. All specimens were amplified in triplicates. Amplification data was normalized against Cyclophilin as endogenous control. Ct values were calculated from automatic threshold. No template controls showed any amplification. Relative expression levels of mRNAs versus control cell lines expressing levels were calculated as 2^(-∆∆Ct).^ Statistical differences between groups were computed by using T-test.

## SUPPLEMENTARY MATERIALS TABLES







## Abbreviations

PDAC: pancreatic ductal adenocarcinoma
FDR: false discovery rate
miRNA: microRNA
NES: normalized enrichment score
FC: fold-change
KO: knock-out
