# Supplementary material for: Deciphering microRNA targets in pancreatic cancer using miRComb R package
Source: Oncotarget. 2018 Jan 8;9(5):6499–517. doi: 10.18632/oncotarget.24034 (PMC5814228; doi:10.18632/oncotarget.24034)
Supplement: Supplementary file 1 [file oncotarget-09-6499-s001.pdf]

Deciphering microRNA targets in pancreatic cancer using miRComb R package

SUPPLEMENTARY MATERIALS

Supplementary Table 1: Clinicopathologic characteristics of individuals included in the study

Characteristic	Patients	
	C (n=3)	PDAC (n=9)
Demographic		
Age, mean (SD)	71.0 (4.0)	69.1 (11.2)
Sex, no.		
Males	2	8
Females	1	1
Pathologic		
Stage, no.		
I		5
II		3
III		1
IV		0

Supplementary Table 2: All miRNA-mRNA interactions in pancreatic cancer found by miRComb (FDR<=0.05)

See Supplementary File 2

Supplementary Table 3: Pathway enrichment analysis for miR-148a miRComb targets by three different methods (GO, KEGG, Reactome)

See Supplementary File 3
